# BASE—high-precision comparisons of the fundamental properties of protons and antiprotons

**DOI:** 10.1140/epjd/s10053-023-00672-y

**Published:** 2023-06-05

**Authors:** B. M. Latacz, B. P. Arndt, B. B. Bauer, J. A. Devlin, S. R. Erlewein, M. Fleck, J. I. Jäger, M. Schiffelholz, G. Umbrazunas, E. J. Wursten, F. Abbass, P. Micke, D. Popper, M. Wiesinger, C. Will, H. Yildiz, K. Blaum, Y. Matsuda, A. Mooser, C. Ospelkaus, W. Quint, A. Soter, J. Walz, Y. Yamazaki, C. Smorra, S. Ulmer

**Affiliations:** 1 RIKEN, Ulmer Fundamental Symmetries Laboratory, 2-1 Hirosawa, Wako, Saitama, 351-0198 Japan; 2 CERN, Esplanade des Particules 1, 1217 Meyrin, Switzerland; 3 Max-Planck-Institut für Kernphysik, Saupfercheckweg 1, 69117 Heidelberg, Germany; 4 GSI-Helmholtzzentrum für Schwerionenforschung GmbH, Planckstraße 1, 64291 Darmstadt, Germany; 5 Institut für Physik, Johannes Gutenberg-Universität, Staudinger Weg 7, 55099 Mainz, Germany; 6 Graduate School of Arts and Sciences, University of Tokyo, 3-8-1 Komaba, Meguro, Tokyo, 153-0041 Japan; 7 Institut für Quantenoptik, Leibniz Universität, Welfengarten 1, 30167 Hannover, Germany; 8 Eidgenössisch Technische Hochschule Zürich, Rämistrasse 101, 8092 Zürich, Switzerland; 9 Physikalisch-Technische Bundesanstalt, Bundesallee 100, 38116 Braunschweig, Germany; 10 Helmholtz-Institut Mainz, Johannes Gutenberg-Universität, Staudingerweg 18, 55128 Mainz, Germany; 11 Heinrich-Heine Universität, Universitätsstraße 1, 40225 Düsseldorf, Germany

## Abstract

**Abstract:**

The BASE collaboration at the antiproton decelerator/ELENA facility of CERN compares the fundamental properties of protons and antiprotons with ultra-high precision. Using advanced Penning trap systems, we have measured the proton and antiproton magnetic moments with fractional uncertainties of 300 parts in a trillion (p.p.t.) and 1.5 parts in a billion (p.p.b.), respectively. The combined measurements improve the resolution of the previous best test in that sector by more than a factor of 3000. Very recently, we have compared the antiproton/proton charge-to-mass ratios with a fractional precision of 16 p.p.t., which improved the previous best measurement by a factor of 4.3. These results allowed us also to perform a differential matter/antimatter clock comparison test to limits better than $3\,$%. Our measurements enable us to set limits on 22 coefficients of CPT- and Lorentz-violating standard model extensions (SME) and to search for potentially asymmetric interactions between antimatter and dark matter. In this article, we review some of the recent achievements and outline recent progress towards a planned improved measurement of the antiproton magnetic moment with an at least tenfold improved fractional accuracy.

**Graphic Abstract:**

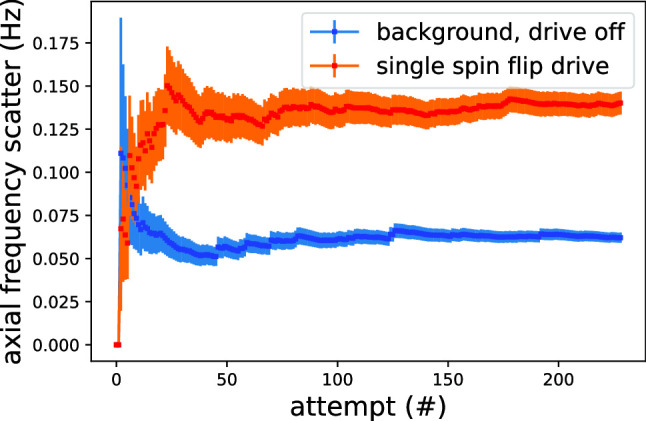

## Introduction

The fundamental charge, parity, time reversal (CPT) invariance [1], which is a combined discrete symmetry transformation, is deeply implemented into the relativistic quantum field theories of the standard model of particle physics. In fact, within the currently available experimental tests of the fundamental interactions, CPT invariance is the only combination of discrete symmetry that is observed as an exact symmetry of nature. Efforts to place quantum field theory on rigorous mathematical grounds [2] have shown that CPT symmetry itself requires only very few general assumptions [3] and has therefore a truly fundamental character. Any local (1), Lorentz and translation covariant field theory (2) that is represented by a reasonably smooth field operator implementation (3), and which has a stable vacuum ground state (4) without momentum and angular momentum (5), conserves CPT symmetry. The CPT operation contains charge conjugation and defines that particle/antiparticle conjugates have identical masses and lifetimes, as well as the same charges and magnetic moments, the latter two with opposite sign. Another consequence of CPT invariance is that conjugate matter and antimatter bound states have exactly the same energy spectrum [3]. Any detected difference in the fundamental properties of matter/antimatter conjugates would challenge the requirements (1) to (5) and would indicate new physics, which inspires experiments that compare the fundamental properties of matter/antimatter conjugates with great precision. For example, electron and positron magnetic moments were compared with a fractional accuracy on the level of $\approx 4\cdot 10^{-12}$ [4], the 1 S/2 S transitions of hydrogen and antihydrogen were compared with a fractional resolution of $2\cdot 10^{-12}$ [5, 6], or the masses of neutral Kaons were investigated with a fractional accuracy of $<10^{-18}$ [7]. In addition to these direct efforts, several model-dependent tests of Lorentz and CPT symmetry [8] have been carried out, for example, by searching for oscillatory structures in co-magnetometer data [9–11], MASER Zeeman transitions [12], and many different other branches of physics [8].

Inspired by the same motivation, our measurements at the BASE experiment [13] at CERN, Geneva, Switzerland, compare the fundamental properties of protons and antiprotons with high precision. Up to now, we have reached a fractional precision of 16 p.p.t. [14] in antiproton/proton charge-to-mass ratio comparisons [15], as well as fractional accuracies of 1.5 p.p.b. [16], and 300 p.p.t. [17] for the magnetic moments of the antiproton and the proton, respectively.

In this paper, we review some of the recent achievements of BASE, with particular focus on a recent differential comparison of matter/antimatter clocks [14] and report on new technological improvements developed to reach fractional uncertainties below $100\,$p.p.t. in the future antiproton, proton, and $\text {H}^{-}$ magnetic moment measurements.

## The BASE experiment

**Fig. 1:**
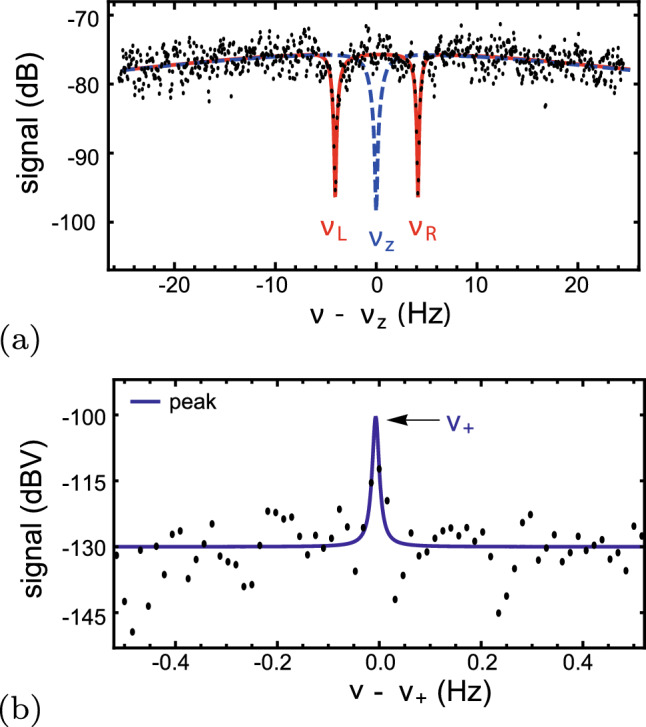
Two methods of measuring the modified cyclotron frequency in the Penning trap **a** sideband method **b** peak method. Figure taken and modified from [14]

The heart of the BASE experiment is a cryogenic four-Penning trap system, made out of gold-plated copper electrodes, enclosed inside a superconducting magnet operated at $\text {B}_{0} = 1.945\,$T. A single particle confined in our trap oscillates with three independent eigenfrequencies, one along the magnetic field lines called the axial frequency at $\nu _{z}\approx 640$ kHz and two in the radial direction, perpendicular with respect to the main axis of the magnetic field—the magnetron mode oscillating at $\nu _{-}\approx 7$ kHz and the modified cyclotron mode at $\nu _{+}\approx 29.641\,$MHz. According to the invariance theorem [18], the root of the squared sum of these frequencies is equal to the cyclotron frequency $\nu _{c}$ of a particle $\sqrt{\nu _+^2 + \nu _z^2 + \nu _-^2} = \nu _c = 1/(2\pi ) \cdot q/m \cdot B_0$. All three frequencies can be measured non-destructively, allowing to reach high measurement stability and excellent data sampling statistics. The axial frequency is measured by picking up the femtoampere currents that are induced in the trap electrodes by an oscillating trapped particle. For signal pickup, superconducting toroidal resonators connected to the trap electrodes are used [19]. Once the particle is in thermal equilibrium with the ultra-sensitive detector, the fast Fourier transform (FFT) signal of the amplified Johnson noise of the superconducting resonator shorted at a frequency equal to the axial frequency of the particle is recorded, resulting in a characteristic dip feature, as shown in blue in Fig. 1a. The frequencies of the radial modes can be measured using *the sideband method* [20], in which classical Rabi oscillations between the axial mode and the radial modes are induced using quadrupolar radiofrequency drives [21], at $\nu _+-\nu _z$ for the modified cyclotron mode and at $\nu _z+\nu _-$ for the magnetron mode. The drive signals modulate the amplitudes of the particle trajectories, resulting in the two sideband dip signals shown in red in Fig$.\,$1a, from which the radial frequency can be calculated. Additionally, the modified cyclotron frequency can be measured using *the peak method * [14, 15], in which the image current from the excited mode is picked up with a dedicated detector that is sensitive at $\nu _+$ [22], resulting in the peak spectrum shown in 1b.

## Proton-to-antiproton charge-to-mass ratio comparison at 16 p.p.t. precision

In [14], BASE published a new measurement of the antiproton-to-proton charge-to-mass (*q*/*m*) ratio $R_{\bar{\text {p}},\text {p}}$. This measurement relies on the comparison of antiproton and H$^-$-ion cyclotron frequencies. By using the negatively charged hydrogen ion H$^-$ as a proxy for the proton, as first applied in [15], the polarity of the trap potentials is conserved, which greatly reduces systematic corrections and uncertainties. As a result, we obtained for the antiproton-to-proton charge-to-mass ratio:

1 $$\begin{aligned} R_{\bar{\text {p}},\text {p}}=\frac{\nu _{c,{\bar{p}}}}{\nu _{c,{p}}}=\frac{(q/m)_{\bar{p}}}{(q/m)_{{p}}} =-1.000~ 000~000 ~ 003(16) \end{aligned}$$

which has an unprecedented precision of 16 p.p.t. [14].

In the related measurement campaign, between December 2017 and May 2019, a total of about 24000 antiproton/H$^-$-cyclotron frequency ratio measurements were accumulated using both the sideband and the peak cyclotron frequency measurement methods. In the implemented measurement protocol [14], a single charge-to-mass ratio comparison required $\approx 240\,$s of measurement time, about 50 times faster than in previous antiproton/proton comparisons [15]. Our recent data sampling campaign improved our previous measurement [23] by a factor of 4.3 and allows to test the standard model with an energy resolution of $2\times 10^{-27}$ GeV, which improved limits on 10 coefficients of the Standard Model extension that characterizes Lorentz- and CPT-violating interactions [8, 24].

The fourfold improvement was achieved due to many technological improvements that were implemented into the experiment, namely new cryostat-to-trap connections and decoupled mechanical support of the experiment, homogenization of the magnetic field, the implementation of a superconducting multi-layer magnetic shielding system [25], and the development of a new highly sensitive tuneable axial detector, which eliminated the main systematic uncertainty of the previous experiment related to the need of re-adjusting the trap voltage separately for the antiproton and the H$^-$-ion.

## First differential test of the clock weak equivalence principle with protons and antiprotons

One of the candidates to explain the matter–antimatter imbalance that is observed on cosmological scales is based on the possibility that the gravitational interaction of antimatter is different than for matter [26]. This hypothesis can be probed by testing the weak equivalence principle (WEP) for antimatter [27]. Although of outstanding interest, precise experimental tests that compare the gravitational behaviour of matter and antimatter are scarce. A test of the WEP has been done for neutral kaons [28], where time variations of the experiment observables were investigated and correlated to changes in astrophysical potentials. In addition, three experiments at CERN—GBAR [29], AEgIS [30], and ALPHA-g [31]—are currently being commissioned to study the ballistic behaviour of antihydrogen in the gravitational field of the Earth. These experiments test the force component of the gravitational interaction tensor and the weak equivalence principle of the free fall (WEP$_{ff}$). In BASE, we contribute to this puzzle by testing the weak equivalence principle by comparing proton and antiproton clocks (WEP$_{cc}$) in the gravitational potential, while the Earth is orbiting around the Sun—the measurement campaign described above provides data sampled over the course of one and a half years.

The gravitational redshift of clocks links their oscillation frequencies to the gravitational potential. In case anomalous gravitational scalar or tensor interactions exist for antimatter [32], in the presence of a gravitational potential proton and antiproton clocks may run at different frequencies [33], making the clock-like antiproton/proton cyclotron frequency comparisons sensitive to the possible WEP$_{cc}$ violations,

2 $$\begin{aligned} \frac{\nu _{c,{{\bar{p}}}}-\nu _{c,{p}}}{\nu _{c}}=\frac{3 \varPhi }{c^2}\left( \alpha _g-1\right) \end{aligned}$$

where $\nu _{c,p,{\bar{p}}}$ are the measured cyclotron frequencies of the proton and the antiproton, $\alpha _g-1$ is a parameter characterizing the strength of the WEP$_\text {cc}$ violation, and $\varPhi$ is the gravitational potential.

Directly using this interpretation together with the value of the gravitational potential of the local supergalactic cluster $\varPhi /c^2=(\text {G M})/(r c^2)=2.99\times 10^{-5}$ [34, 35], our measurements constrain the WEP$_\text {cc}$ violating parameter $|\alpha _{g,G}-1| < 1.8 \times 10^{-7}$, which improves the previous limit by a factor of 4.3. However, this direct comparison is model-dependent, as it requires certain assumptions about the value of the gravitational potential, and does not consider contributions by dark energy.

**Fig. 2:**
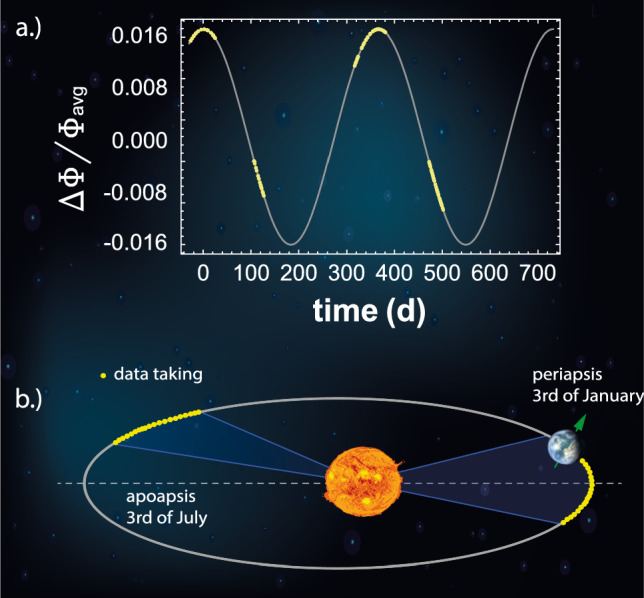
Variation of the gravitational potential in the BASE laboratory sourced by the elliptical orbit of the Earth around the Sun. The yellow scatter points represent the data-taking windows, in plot **a** for the fractional change of the gravitational potential and in plot **b** for the Earth’s trajectory around the Sun. Figure similar to [14]

After reaching a sampling rate of the cyclotron frequencies of a proton and an antiproton of only 240 s per ratio determination, and a parts-per-billion shot-to-shot stability of the experiment, we performed a model-independent test of WEP$_\text {cc}$ based on the difference of the gravitational potential resulting from the elliptical trajectory of the Earth around the Sun, see Fig. 2. As indicated in yellow, we covered 80 % of the peak-to-peak variation in the gravitational potential, which allowed us to constrain the WEP$_{cc}$ violating parameter to $|\alpha _{g,D}-1|<0.030$ (60 % C.L.). This constitutes the first model-independent confirmation of the universality of clocks (WEP$_{cc}$) comparing matter–antimatter conjugates, which is an experimental consequence of local time invariance—an ingredient of the weak equivalence principle (WEP). The presented result is at the anticipated precision level of experiments that aim at testing the WEP$_{ff}$ [29–31]. The detailed interpretation of the effective parameter $\alpha _{g,D}$ has to include the structure of the antiproton, as the valence antiquarks constitute $\approx 10\,$MeV/c$^2$ of the mass of the antiproton, as described in, for example, [36, 37].

## Towards a 100 p.p.t. antiproton/proton/hydrogen ion $\text {H}^{-}$ magnetic moment measurement

The standing record regarding the precision of the magnetic moment measurement of the antiproton has a fractional accuracy of 1.5 p.p.b. and was published in 2017 [16] by our collaboration. In parallel to the charge-to-mass ratio measurement [14], we developed a considerably upgraded experimental setup and improved measurement methods, with the goal to reach a fractional accuracy of $<100\,$p.p.t. for antiproton, proton, and hydrogen ion $\text {H}^{-}$ magnetic moment measurements.

The *g*-factor of the antiproton, i.e. the magnetic moment in units of the nuclear magnetron $\mu _{N}$, can be measured by evaluating the ratio of the Larmor frequency $\nu _{L}$ and the cyclotron frequency $\nu _{c}$ of a particle $g_{{{\bar{p}}}}/2 = -\mu _{{\bar{p}}}/\mu _{N} = \nu _{L} / \nu _{c}$. The cyclotron frequency is measured by straightforward image-current detection [14], while for the measurement of the Larmor frequency the continuous Stern–Gerlach effect [38] is applied, which in ultra-strong magnetic inhomogeneity couples the magnetic moment of the particle to its measurable axial oscillation frequency and allows to achieve a single-particle quantum-transition spectroscopy. Due to the small absolute magnitude of the proton/antiproton magnetic moments, the axial frequency jumps created by spin transitions are challenging to resolve. To make them visible, we superimpose on one of our traps a magnetic bottle with a strength of $265.7 (3.1)\,$kT/m$^2$. This magnetic bottle does couple not only the spin magnetic moment but also orbital magnetic moments to the axial frequency $\nu _z$, making the experiment extremely sensitive to parasitic noise on the trap electrodes, which causes heating of the cyclotron mode and thus induces axial frequency fluctuations. We have shown that the heating rate scales linearly as a function of the temperature of the modified cyclotron mode $T_+=E_+/k_B$ [39], the single spin-flip resolution that is required in our multi-trap experiments is reached at $E_+/k_B<200\,$mK. Therefore, a measurement of the Larmor frequency requires a cold particle, while each cyclotron frequency measurement thermalizes the particle to at least 320 K. As in the 2017 measurement campaign the sub-thermal cooling [40] of an antiproton to temperature $T_{+}<200$ mK took on average about 15 h, a novel two-particle technique was invented [16] which greatly improves the data accumulation rate. In this two-particle scheme, a cold particle is used to measure the Larmor frequency, while the second one probes the magnetic field (cyclotron frequency). However, due to the uncertainty of the measurement of the particles temperatures and present fluctuations and inhomogeneities in the magnetic field of the trap, the two particles probe effectively slightly different average magnetic fields, which lead to the dominant systematic uncertainty of this measurement of $(\varDelta g / g)_{\text {drive}} = 0.97\,$p.p.b. Also, in the 2017 campaign, the linewidth of the Larmor resonance was deliberately saturated to 15 p.p.b. to make the measurement robust with respect to magnetic field fluctuations imposed by the antiproton decelerator facility. Measuring at higher magnetic field stability and thus with lower drive amplitude will reduce the line width and lead to higher precision. Below, we will describe the layout and status of a recently upgraded magnetic moment measurement setup at BASE, which was designed to decrease systematic uncertainties and to increase the *g*-factor measurement precision to be able to reach our defined new goal.

### New trap stack

**Fig. 3:**
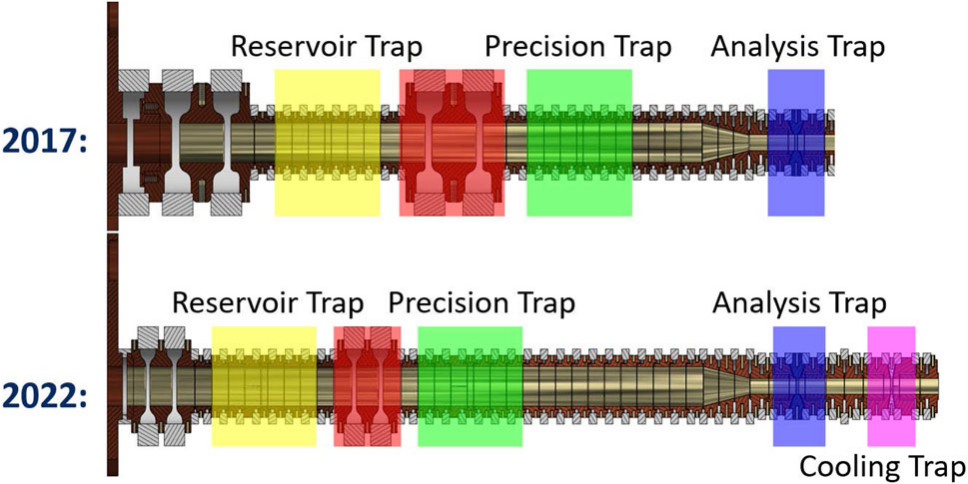
Comparison of the layout of the four-Penning trap system used in 2017 and in the 2022 measurement campaigns. The red box indicates a parking electrode

The four-Penning trap system of the BASE experiment, shown in Fig. 3, is constituted by the reservoir trap (RT) for storing and catching antiprotons [41], the precision trap (PT) with a homogeneous magnetic field in which the precision measurements of the Larmor and cyclotron frequencies take place, and the analysis trap (AT) where the large magnetic bottle is superimposed to identify the particle’s spin state via the continuous Stern–Gerlach effect. Additionally, in 2022 we have implemented a cooling trap, which is a device dedicated for efficient sub-thermal cooling of an antiproton, to efficiently prepare particles with temperatures below the direct spin-state detection threshold $T_+<200\,$mK.

**Table 1:** Residual linear ${B}_{1}$ and quadratic ${B}_{2}$ components of the magnetic field in the precision trap in the 2017 setup, in the 2022 setup, and after tuning the magnetic field with the superconducting shimming system

	2017	2022 trap stack	2022 tuned
${B}_{1}$ (T/m)	0.0712(4)	0.0270(7)	0.0162(4)
${B}_{2}$ (T/m$^2$)	2.7(3)	0.1298(8)	0.0003(3)

The main systematic uncertainty of the 2016/2017 measurement was caused by a residual magnetic field inhomogeneity along the magnet axis in the PT $\text {B}_{PT}\approx \text {B}_{0}+ \text {B}_{1}\cdot z+\text {B}_{2} \cdot z^{2}$ imposed by the far field of the ferromagnetic ring electrode of the AT. To decrease this effect, we have redesigned the trap, decreased the size of the high voltage catching electrodes of the RT, and increased the distance between the AT and the PT by adding transport electrodes. This allowed to decrease the values of $\text {B}_{1}$ and $\text {B}_{2}$ in the PT by a factor of 2.6 and 21, respectively; see Table 1 for exact values.

Additionally, we implemented a superconducting shimming system around the precision trap to be able to further tune the values of $\text {B}_{1}$ and $\text {B}_{2}$. First results show the possibility to reduce the $\text {B}_{2}$ component of the PT by more than a factor of $10\,000$ and to tune the $\text {B}_{1}$ part down by another factor of 2, which will practically eliminate the dominant systematic uncertainties of the 2017 *g*-factor measurement [16].

### Improved cyclotron frequency stability

Measuring the *g*-factor requires the simultaneous measurement of both the Larmor frequency and simultaneously the cyclotron frequency with high accuracy. In 2017, the Larmor resonance was saturated to a width of 15 p.p.b. making the spin-flip spectroscopy robust with respect to frequency fluctuations caused by changing magnetic fields inside the AD/ELENA facility. To reduce this effect, we installed a multi-layer superconducting shielding system, which decreases some external magnetic field disturbances by up to a factor of $225\pm 15$ [25]. We expect that this upgrade will allow us to reduce the width of the Larmor resonance at least to the 1 p.p.b. level, as earlier demonstrated at our experiment BASE Mainz, Germany [17].

**Fig. 4:**
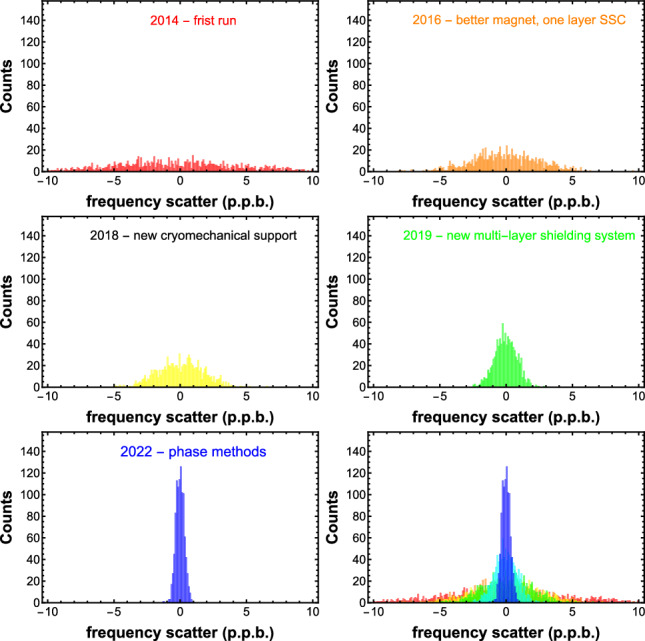
Improvement in the cyclotron frequency scatter $\sigma (\varDelta \nu _{+})$ measured in the BASE experiment throughout the years

Another aspect is the fluctuation of the cyclotron frequency measurements, whose history is presented in Fig$.\,$4. In 2014, BASE started with a shot-to-shot cyclotron frequency scatter of $\sigma (\varDelta \nu _{+})\approx 5.5\,$p.p.b. [23]. In the following years, the experiment was systematically improved, first by implementing a new superconducting magnet (2015) and a single-layer self-shielding coil system [42] and later by revising the cryomechanical support (2018), reaching in 2019 with the added multi-layer system a cyclotron frequency fluctuation of $\sigma (\varDelta \nu _{+})\approx 1.8\,$p.p.b., which was one of the key improvements for the 16 p.p.t. charge-to-mass ratio comparison. Based on these experimental upgrades and inspired by the goal to further improve the resolution of our *g*-factor measurements, we have implemented peak and phase-sensitive frequency measurement methods. Combined with the multi-layer superconducting shielding system [25], we have reached with these methods frequency scatters of $\sigma (\varDelta \nu _{+})\approx 520(50)\,$p.p.t. for peak-based measurements and $\sigma (\varDelta \nu _{+})\approx 280(20)\,$p.p.t. for phase-sensitive measurements, at a shot-to-shot sampling rate of 1/(265 s) [14].

### Detection of spin flips in the analysis trap

**Fig. 5:**
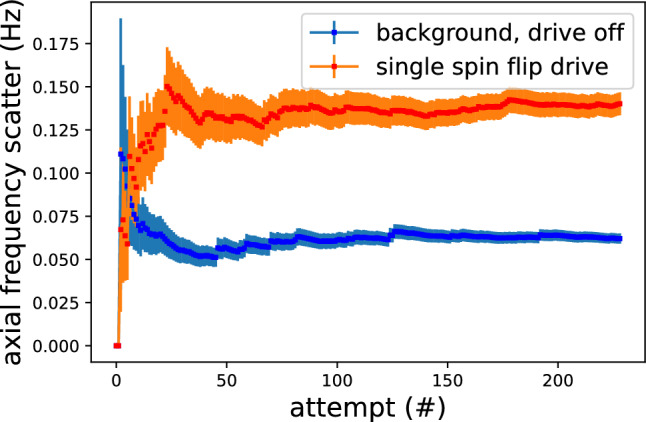
Evolution of the mean values of the frequency scatter for no-drive (blue) and spin-flip drive (red) as a function of attempts

The most important part for the planned improved proton/antiproton magnetic moment measurement is the non-destructive detection of single nuclear spin-quantum transitions in the analysis trap. Here, we report on the unambiguous observation of such spin transitions in the newly implemented trap system, for the first time after 6 years [43]. To detect these transitions, we first prepare a single particle in the AT and carefully optimize the electrostatic potential of the trap, which provides high single-particle detection signal-to-noise ratio and enables axial frequency measurements at high resolution. With this particle, we measure the axial frequency, irradiate a spin-flip drive, and measure the axial frequency again. Afterwards, we evaluate the axial frequency scatter $\varXi _{SF}$, which is the root-mean-square value of subsequently recorded axial frequency differences, with and without the spin-flip drive tuned to the Larmor resonance. The mean scatter of the axial frequency with resonant spin-flip drive on is equal to [44]

3 $$\begin{aligned} \varXi _{SF} \approx \sqrt{ \varXi _{back}^{2} + P_{SF}\cdot (\varDelta \nu _{z,SF})^{2} } \end{aligned}$$

where $P_{SF}$ is the spin-flip probability, $\varXi _{back}$ is the background axial frequency scatter with the drive switched off, and $\varDelta \nu _{z,SF}=173(2)$ mHz is the change of the axial frequency caused by a single spin flip. A measurement of the cumulatively determined axial frequency scatter for both no-drive and spin-flip drive on is shown in Fig. 5, where we achieve a spin-flip probability of $P_{SF}=54(6)$ % with a background scatter of 62(1) mHz, which unambiguously demonstrates that with the newly developed experiment a spin-state detection fidelity similar to the one achieved in 2017 was reached. With the new experimental setup, we have used the AT also to detect single-spin transitions that were induced in the PT, similar to the work described in [45]. The noise and experiment optimization work is currently ongoing to further decrease the background axial frequency scatter, which would allow us to further increase the data sampling rate of the experiment.

## Summary and outlook

Very recently, the BASE collaboration has published the most stringent test of CPT invariance in the baryonic sector of the standard model by measuring the antiproton-to-proton charge-to-mass ratio with a fractional precision of 16 p.p.t. [14]. This measurement improved the previous best result [23] by a factor of 4.3, and improved by the same factor constraints on 10 exotic interactions described within the standard model extension [8]. Moreover, this 1.5-year-long measurement campaign allowed us to perform the first differential, and thus model independent, test of the weak equivalence principle of clocks for antimatter, showing no violation at the level of 3 %. Secondly, BASE is currently commissioning an upgraded experimental setup with the goal to measure the *g*-factor of the antiproton, the proton, and for the first time also of the hydrogen ion $\text {H}^{-}$ with the ultimate goal to reach a fractional uncertainty of about 100 p.p.t. For this, we developed a new trap system which incorporates an upgraded superconducting shielding and local magnetic shimming system, to stabilize and homogenize the magnetic field of the trap. With this upgraded multi-Penning trap setup, we have reached in cyclotron frequency measurements a shot-to-shot stability of better than 500 p.p.t. and have considerably reduced residual magnetic gradients in the measurement trap. We observed single-proton spin-quantum transitions in the AT with a background axial frequency scatter of only 62(1) mHz, which constitutes a crucial milestone towards the planned improved magnetic moment measurements.

## Data Availability

This manuscript has no associated data or the data will not be deposited. The data presented in this paper and the related analysis code will be made available on reasonable request.
